# Improved Li storage performance in SnO_2_ nanocrystals by a synergetic doping

**DOI:** 10.1038/srep18978

**Published:** 2016-01-06

**Authors:** Ning Wan, Xia Lu, Yuesheng Wang, Weifeng Zhang, Ying Bai, Yong-Sheng Hu, Sheng Dai

**Affiliations:** 1 Key Laboratory of Photovoltaic Materials of Henan Province and School of Physics & Electronics, Henan University, Kaifeng 475004, PR China; 2 Materials Engineering, McGill University, Montréal (Québec) H3A 0C5, Canada; 3 Institute of Physics, Chinese Academy of Sciences, Beijing 100190, PR China; 4 Chemical Sciences Division, Oak Ridge National Laboratory, Oak Ridge, TN 37831, USA

## Abstract

Tin dioxide (SnO_2_) is a widely investigated lithium (Li) storage material because of its easy preparation, two-step storage mechanism and high specific capacity for lithium-ion batteries (LIBs). In this contribution, a phase-pure cobalt-doped SnO_2_ (Co/SnO_2_) and a cobalt and nitrogen co-doped SnO_2_ (Co-N/SnO_2_) nanocrystals are prepared to explore their Li storage behaviors. It is found that the morphology, specific surface area, and electrochemical properties could be largely modulated in the doped and co-doped SnO_2_ nanocrystals. Gavalnostatic cycling results indicate that the Co-N/SnO_2_ electrode delivers a specific capacity as high as 716 mAh g^−1^ after 50 cycles, and the same outstanding rate performance can be observed in subsequent cycles due to the ionic/electronic conductivity enhancement by co-doping effect. Further, microstructure observation indicates the existence of intermediate phase of Li_3_N with high ionic conductivity upon cycling, which probably accounts for the improvements of Co-N/SnO_2_ electrodes. The method of synergetic doping into SnO_2_ with Co and N, with which the electrochemical performances is enhanced remarkably, undoubtedly, will have an important influence on the material itself and community of LIBs as well.

High-energy lithium-ion batteries (LIBs) have already played a crucial role in the development of consumer electronics, electric vehicles, and grid-scale stationary energy storage. As expected, there has been a growing demand for LIBs with improved electrochemical performance with regard to higher energy density, longer cycle life and extreme safety^1^,^2^,^3^,^4^,^5^,^6^,^7^. When it refers to practical battery applications, the energy density (per weight or volume), cycle life, and safety issues, the major focuses of recent battery research, are also the most important factors considered for next-generation high-performance LIBs. Currently, graphite is a commonly used anode material in commercial LIBs. However, it is far from ideal one due to its low initial coulombic efficiency, large amount of solid electrolyte interphase (SEI) formation, and asymmetric discharge/charge process^8^,^9^. Compared with other anode candidates, graphite also lacks a capacity advantage (*vs.* crystal/amorphous silicon), the rate capability (*vs.* Li_4_Ti_5_O_12_ spinel), and some other favorable characteristics. Therefore great effort is being devoted to balancing the major requirements of the qualified anode for LIBs^9^.

According to various reports, metal-oxide anodes demonstrate attractive prospects after effective modifications. Tin dioxide (SnO_2_), particularly, has been widely investigated for its lithium (Li) storage mechanism of an initial phase conversion reaction followed by a Li-Sn alloying reaction to deliver a theoretical specific capacity of 1494 mAh g^−1^ (SnO_2_ + 4 Li^+^ + 4 *e*^*−*^ → 2Li_2_O + Sn; Sn + 4.4 Li^+^ + 4.4 *e*^*−*^ ↔ Li_4.4_Sn)^10^,^11^,^12^. Although the specific capacity is around four times larger than that of a graphite anode, SnO_2_ electrode suffers from poor cycleability, with obvious capacity loss after repeated cycling. This capacity loss is mainly caused by the severe particle pulverizations that is triggered by a more than 250% volume change (expansion and shrinkage) upon lithiation/delithiation^13^,^14^. Upon long cycles, the pulverization of SnO_2_ particles is directly associated with a decay in capacity, as uncontrolled volume variations lead to cracking of the electrode, which in turn causes a loss of direct contact with current collector and the electronic conductivity network. This volume change also induces additional growth of SEI layers on the naked SnO_2_ surface, thereby introducing a corresponding mechanical instability^15^,^16^. Moreover, the poor cycleability inevitably results in serious capacity fading upon cycling, especially at high current densities^17^. Thus it is essential to improve the cycleability of SnO_2_ electrodes by suppressing particle pulverization and volume change, and further enhancing the electronic conductivity network to ensure superior electrochemical performances for use in LIBs.

Among the available techniques, element doping is an effective method that works well to optimize the electrochemical performance of SnO_2_ electrodes^18^,^19^,^20^,^21^. Recently, cobalt (Co) has been successfully doped into the SnO_2_ lattice and it was found that the doped samples exhibit a volume buffering effect (*i.e.*, being able to accommodate a large volume change) and enhanced electronic conductivity^18^,^19^. In work by Chien-Te *et al.*^18^, a Co-doped SnO_2_ electrode released a specific capacity of only about 530 mAh g^−1^ after 50 cycles, much lower than its theoretical value (for alloying/de-alloying reaction ~782 mAh g^−1^ and for alloying/de-alloying and conversion reactions, ~1494 mAh g^−1^). On the other hand, it is well established that nitrogen (N)-doped SnO_2_ exhibits desirable enhanced electrical and optical properties, which could be attributed to greatly increased electronic conductivity^20^,^21^. Therefore, synergetic doping of Co and N into an SnO_2_ sample should take advantage of the enhanced volume tolerance as well as good electronic conductivity, which would endow the SnO_2_ electrode with better Li storage and transport behaviors.

This article details the application of a hydrothermal method to prepare nanocrystal SnO_2_ and Co-doped SnO_2_ (Co/SnO_2_) samples. After the Co-doped sample was synthesized, it was further thermally treated under N_2_ atmosphere to obtain Co and N co-doped SnO_2_ (Co-N/SnO_2_), which has rarely been reported in previous literatures. All of the samples are employed as anodes for cycling tests. It is found that the Co and N co-doped material exhibits remarkably improved electrochemical properties as a result of the synergetic contributions from the Co and N. Recently, as demonstrated by our group, Cu-N and Ni-N co-doped SnO_2_ also shown enhanced electrode performances^22^,^23^. However, the improvement mechanism has not been intensively explored yet. In this work, not only the electrochemical performance influence of Co-N co-doping on SnO_2_ are extensively studied, more significantly, the intrinsic mechanism is also discussed and revealed. This work paves the way to fully understanding the Li storage and transport mechanism in this important material. Further, it also sheds light on novel structural design and composition-modulated synthesis of electrode materials in advanced LIBs.

## Results and Discussion

### Morphology and structure analysis

**Figure 1:**
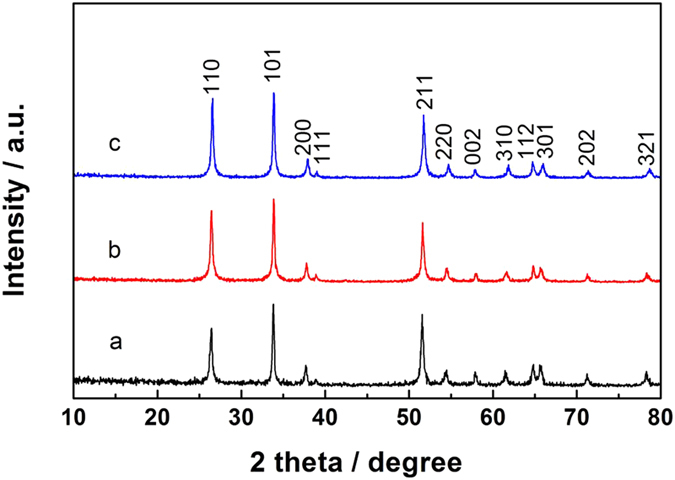
XRD patterns of the as-prepared samples. (**a**) phase-pure SnO_2_, (**b**) Co/SnO_2_ and (**c**) Co-N/SnO_2_ nanocrystals. The prominent peaks of three samples can be easily identified as the tetragonal phase of SnO_2_ (JCPDS card no.01-0657).

Figure 1 shows the XRD patterns of as-prepared phase-pure SnO_2_, Co/SnO_2_, and Co-N/SnO_2_ nanocrystals. It can be observed that the samples are well-crystallized, and all the diffraction peaks can be indexed to the tetragonal rutile SnO_2_ (JCPDS No. 01-0657). No diffraction peak of impurity appears before/after doping, suggesting that the doping does not impact the phase and structure of the obtained SnO_2_ samples. The lattice parameters and grain sizes of SnO_2_, Co/SnO_2_ and Co-N/SnO_2_ samples are calculated using Scherrer’s formula as shown in Table 1. It is clear that the lattice constants of the three samples remain almost the same in the framework of rutile structure before and after Co and N co-doping. This is also an important implication of the successful doping of Co and N ions into rutile SnO_2_ structure, partially because the ionic radius of Co^2+^ (0.0745 nm) is very close to that of Sn^4+^ (0.0690 nm), as is also the case for O^2−^ (0.142 nm) and N^3−^ (0.142 nm) ions^24^. As revealed in Table 1, the grain sizes of the three as-prepared samples are 34.0, 35.6, and 39.2 nm, respectively. It indicates that Co doping does not change the crystalline size of SnO_2_ grains apparently, whereas the process of N doping accelerates its growth as a result of the heat treatment in the N_2_ atmosphere.

Raman spectroscopy is a more sensitive probe of structural distortion and lattice symmetry in solids, especially for the short-range and long-range ordering of materials^17^. Figure S1 (Supporting Information) displays the Raman spectra of the pure SnO_2_, Co/SnO_2_ and Co-N/SnO_2_ samples. Three vibration peaks located at 472, 630, and 774 cm^−1^ correspond to the E_g_, A_g_, and B_2g_ modes of rutile SnO_2_, respectively; and the other three vibrations at 357 (IF), 440 (N_1_) and 571 (P) cm^−1^ relate to its surfactants^25^. It is reported that the intensity of P peak decreased with the growth of SnO_2_ grains^23^. From Figure S1, the intensity ratio of A_1g_/P in the Co-N/SnO_2_ sample is higher than those of the pure SnO_2_ and Co/SnO_2_ samples, indicating the growth in grain size after Co and N co-doping into the SnO_2_ sample, in line with the trend derived from XRD as shown in Table 1. Additionally, the fitting results give the widths (full-width at half-maximum, FWHM) of 24, 22, and 16 cm^−1^ for the A_1g_ mode of the pure SnO_2_, Co/SnO_2_, and Co-N/SnO_2_ samples, respectively, which means that the Co and N co-doping sample displays better crystallinity compared with the pure SnO_2_ sample.

**Figure 2:**
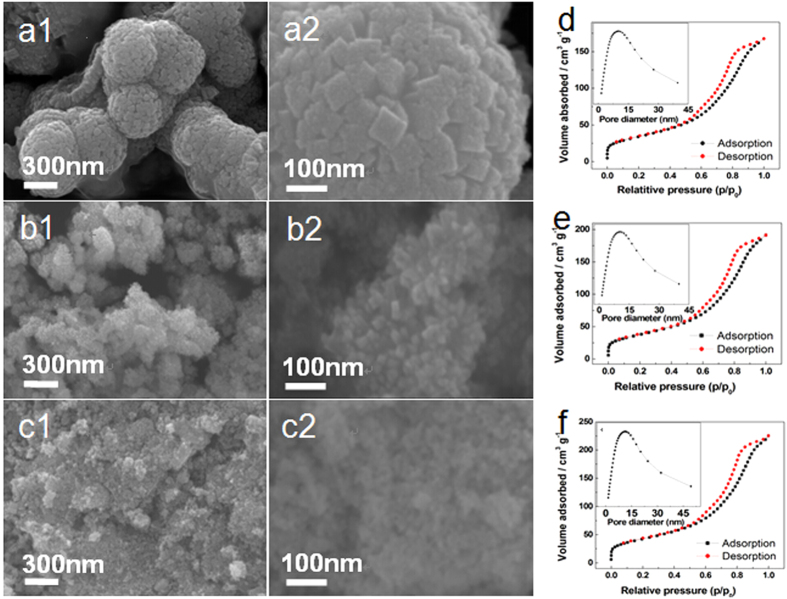
Surface morphologies and BET analysis of the as-prepared samples. SnO_2_ (**a1,a2**), Co/SnO_2_ (**b1,b2**) and Co-N/SnO_2_ (**c1,c2**) samples. N_2_ adsorption-desorption isotherms measured at 77 K for the as-prepared pure (**d**) SnO_2_, (**e**) Co/SnO_2_, and (**f**) Co-N/SnO_2_ samples. The inset is the BJH pore-size distribution of the corresponding materials.

FESEM images of SnO_2_, Co/SnO_2_, and Co-N/SnO_2_ samples are demonstrated in Fig. 2. The three as-prepared samples are clearly composed of nano-sized SnO_2_ particles of less than 100 nm. Pure SnO_2_ demonstrates a peculiarly hierarchical structure with an average diameter of 500 nm, which is agglomerated by initial nano-cubes (with side length of about 80 nm). Note that the Co doping and Co/N co-doping process changes the morphology of SnO_2._ As clearly observed in Fig. 2b,c, Co/SnO_2_ and Co-N/SnO_2_ samples are composed of homogeneously distributed nanoparticles with a similar size of about 30 ~ 40 nm, much smaller than that of the pure SnO_2_ sample (~80 nm). This reduction in particle size will effectively shorten the diffusion path of Li^+^ during electrochemical cycling and thus facilitates a fast charge/discharge process. In addition, the smaller particle size is believed to be beneficial in releasing the interior stresses of SnO_2_ particles and alleviating the influence of volume change upon lithiation/delithiation. The EDAX spectra and element mapping images of the Co/SnO_2_ and Co-N/SnO_2_ samples are demonstrated in Figure S2 (Supporting Information), which not only proves the existence of Co (and N) in the corresponding materials, but also indicates a homogeneous distribution of the doped elements.

The textural properties of pure SnO_2_, Co/SnO_2_, and Co-N/SnO_2_ samples were determined by N_2_ adsorption spectroscopy. Figure 2d,e, and f depict the N_2_ adsorption/desorption isotherms of the phase-pure matrix, as well as those of the composite materials and the corresponding pore size distributions determined by the Barrett-Joyner-Halenda (BJH) model (inset Fig. 2d–f). The isotherms are of type IV adsorption and exhibit H4 hysteresis characterized by a well-defined and steep N_2_ uptake step in a wide relative pressure (P/P_0_) range of 0.60 – 0.90, which is the result of capillary condensation in mesoporous materials. Moreover, the increase in the amount of nitrogen adsorbed at low pressures (P/P_0_ = 0 – 0.1) indicates the presence of nanopores. The BET specific surface areas of the pure SnO_2_, Co/SnO_2_, and Co-N/SnO_2_ samples are respectively 123, 130, and 139 m^2^ g^−1^, indicating an increasing tendency after Co and N doping. Additionally, the BJH pore diameters for the three samples are determined to be 8.7, 8.9, and 9.1 nm, respectively (in Supporting Information Table S1). It can be seen that the as-prepared Co-N/SnO_2_ nanostructure exhibits increases in both BET surface area and pore diameter with respect to pristine SnO_2_ and Co/SnO_2_ samples, which are indeed beneficial for electrolyte penetration and electrode kinetics during cycling.

**Figure 3:**
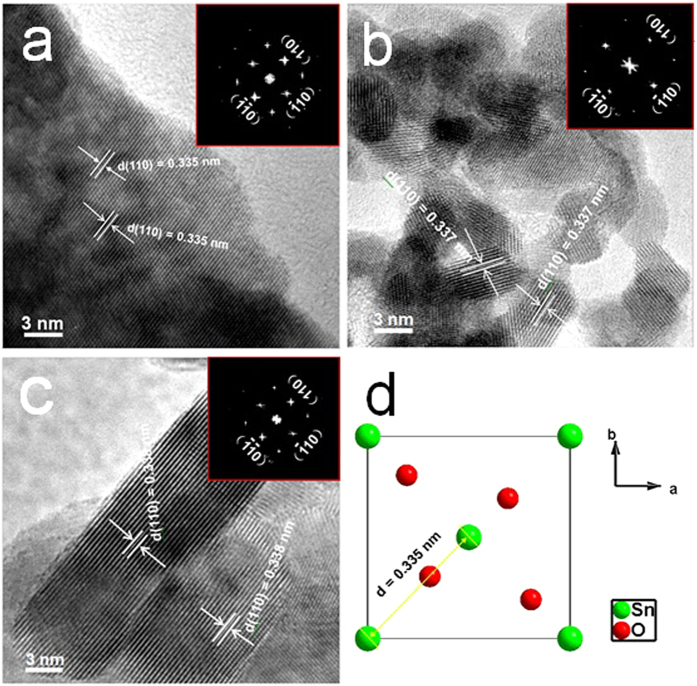
HRTEM images of pure SnO_2_, Co/SnO_2_, and Co-N/SnO_2_ nanoparticles. (**a–c**) the (110) lattice stripes with *d*-spacings of 0.335, 0.337, and 0.338 nm and the inset are the SAED patterns of the pristine and doped SnO_2_ samples. (**d**) Schematic illustration on the lattice structure of rutile SnO_2_ is shown for comparison.

HRTEM images of pristine, Co/SnO_2_, and Co-N/SnO_2_ nanoparticles are displayed in Fig. 3. From the HRTEM images, the SnO_2_ nanocrystals exhibit the homogenous particle sizes and visible lattice stripes. In Fig. 3a,c, the sharp (110) lattice stripes correspond to *d*-spacings of 0.335, 0.337, and 0.338 nm of rutile SnO_2_ lattice at the [001] zone axis, which is consistent with the *d*-spacing of a rutile SnO_2_ (110) plane shown in Fig. 3d. No other planes or *d*-spacing values are distinctly detected to be assigned to the impurity phases. These findings are further confirmed by the selected area electron diffraction (SAED) (shown in the inset), which demonstrates the regular electron diffraction patterns of pure/doped SnO_2_ samples at the [001]* zone axis. From Fig. 3, it can be seen that there is almost no difference in the structure of rutile SnO_2_ before/after Co and Co/N co-doping, and no obvious impurity phases can be observed either in the bulk or on the surface, which is in good agreement with XRD measurements, as shown in Fig. 1.

**Figure 4:**
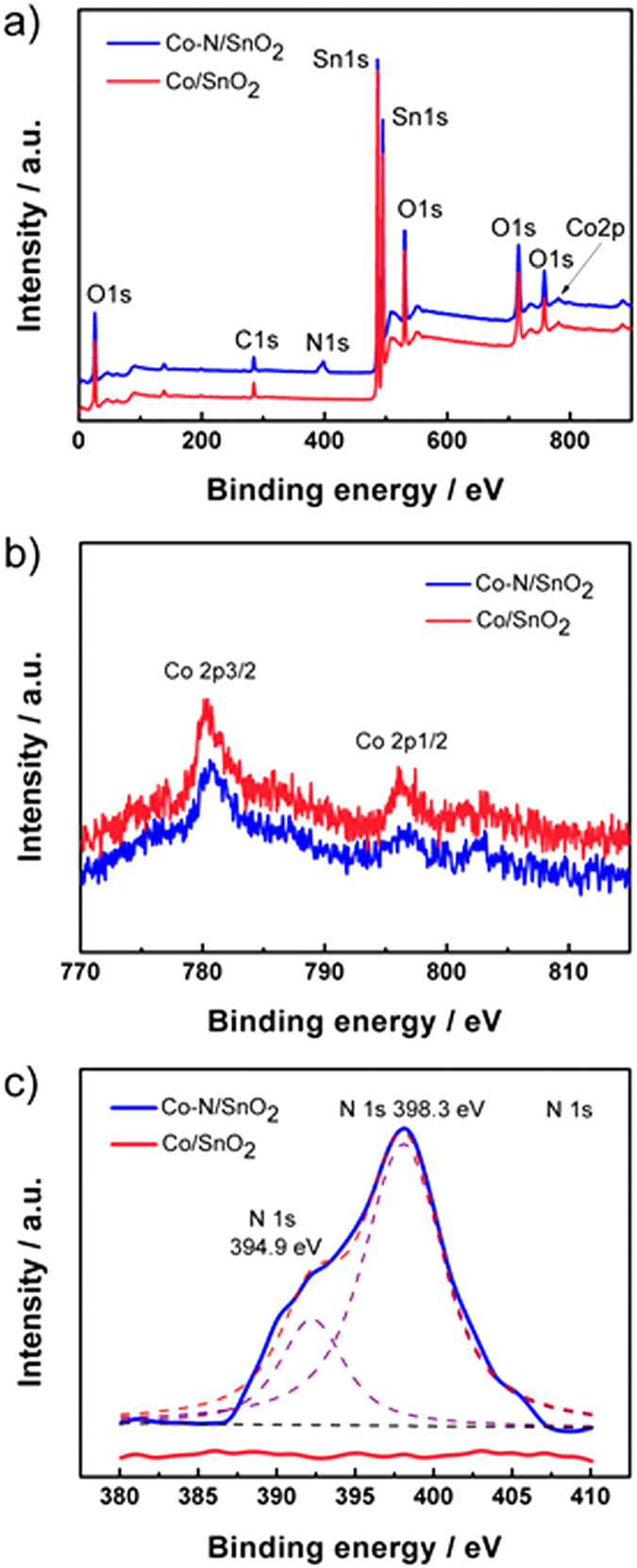
XPS spectra of Co/SnO_2_ and Co-N/SnO_2_ samples: (a) survey profiles and comparisons of Co 2p (b) and N 1s (c) signals.

The doping samples are further verified through XPS analysis. Figure 4a shows the XPS survey spectra of the Co/SnO_2_ and Co-N/SnO_2_ samples, from which the XPS signals of Co and a small amount of N can be observed unambiguously for Co-N/SnO_2_. The binding energies of Co 2p for Co/SnO_2_ and Co-N/SnO_2_ samples are compared as shown in Fig. 4b, where the Co 2p_3/2_ (780.5 eV) and Co 2p_1/2_ (796.2 eV) XPS binding energies for the two samples are detected at almost the same positions, confirming the presence of the same chemical environment Co^2+^ ions in the as-prepared samples (here, Co^2+^ ions substituting for Sn^4+^ ion as 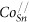)^26^,^27^. This result also implies that doping N into the SnO_2_ lattice does not influence the chemical states of Co^2+^ ions in Co/SnO_2_ samples. On the other hand, Co^2+^ ions are well-known to be electrochemically inactive in the voltage range of 0 to 3 V^28^. Therefore, the presence of inactive Co^2+^ ions should be helpful in stabilizing the structure and affording the volume change for a SnO_2_ electrode upon cycling. The binding energies of N 1s are detected at 394.9 and 398.3 eV on sample Co-N/SnO_2_ (Fig. 4c), which clearly demonstrates the successful incorporation of N species in SnO_2_ crystal structure^29^. This nitrogen doping behavior is presumably ascribed to the decomposition of nitrate at 500 °C during sample annealing^30^, in consistence with the N element mapping result as shown in Figure S2k. The existence of N in the Co-N/SnO_2_ nanocrystals will be beneficial in facilitating electronic conductivity^20^,^21^, which will be discussed in the following sections.

### Electrochemical performances

**Figure 5:**
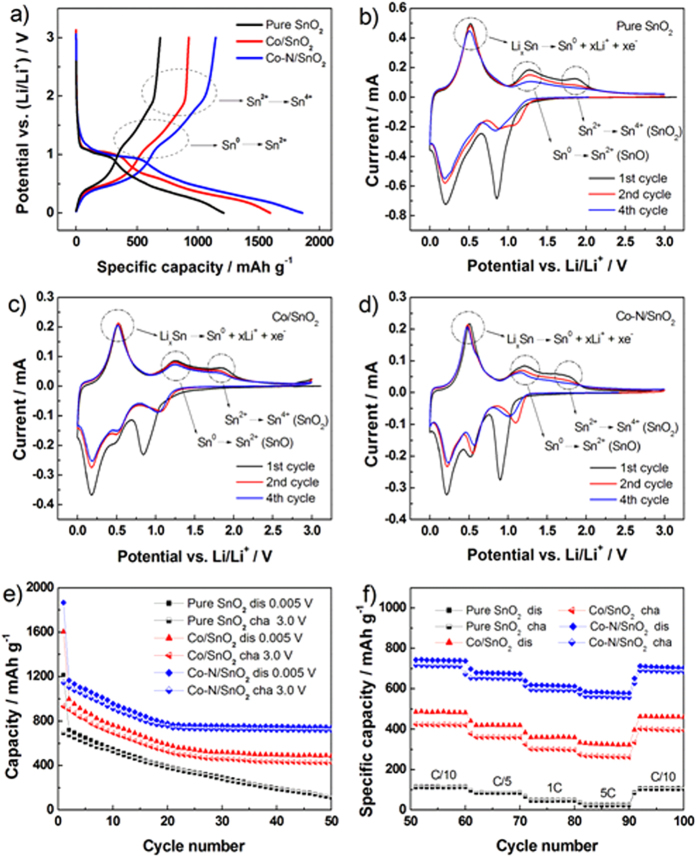
Comparison of electrochemical performances of the as-prepared samples. (**a**) Charge-discharge curves for pure SnO_2_, Co/SnO_2_ and Co-N/SnO_2_/Li cycled between 3.0 and 0.005 V at a rate of 0.1 C (1 C = 782 mA g^−1^). CV curves of the pure SnO_2_ (**b**), Co/SnO_2_ (**c**) and Co-N/SnO_2_ (**d**) electrodes at a scanning rate of 0.10 mV s^−1^. Cycling performances at 0.1 C (**e**) and rate capabilities (**f**) of SnO_2_, Co/SnO_2_ and Co-N/SnO_2_ electrodes (“dis” and “cha” represent the states of discharge and charge upon cycling).

Figure 5a shows the initial charge/discharge profiles of pure SnO_2_, Co/SnO_2_, and Co-N/SnO_2_ electrodes between 0.005 and 3.0 V under a current density of 0.1 C (1 C = 782 mA g^−1^). In Fig. 5a it appears that, of the three electrodes, the Co-N/SnO_2_ electrode demonstrates the highest initial charge and discharge capacities, which could be attributed to its improved crystallinity and good electronic conductivity^20^,^21^. On the other hand, the irreversible capacities for pure SnO_2_, Co/SnO_2_, and Co-N/SnO_2_ are respectively 523, 653, and 709 mAh g^−1^ in the first cycle. One important reason for the large capacity loss of the Co-N/SnO_2_ electrode lies in the increased specific surface area, as shown in Table S1, which will inevitably result in excess formation of SEI film on the electrode surface^31^. Although the three samples show similar trends in initial discharge/charge profiles, the Co-N/SnO_2_ electrode exhibits two plateau-like discharge profiles at around 0.94 and 0.23 V, which can be separately ascribed to the structural destruction of SnO_2_ at ~1.0 V *vs*. Li and formation of an intermediate SnO phase (2Li + SnO_2_ → Li_2_SnO_2_ (SnO + Li_2_O)). Subsequently, intermediate-phase SnO is further reduced to metallic Sn^0^ nanoparticles (2Li + Li_2_SnO_2_ → 2Li_2_O + Sn) and then the alloying reaction of Sn^0^ with Li occurred^32^,^33^. These processes have clear reduction peaks in the cathodic segment of the CV for Co-N/SnO_2_ electrode, namely, the sharp peaks at about 0.90 and 0.21 V. In the initial charging process, the oxidation of Sn^0^ to Sn^2+^ (SnO) and further to Sn^4+^ (SnO_2_) could be inferred in combination with CV characterization, which will be discussed later. From Fig. 5a it can be clearly seen that the plateau capacities at ~0.90 V are around 290, 380, and 540 mAh g^−1^ for pristine, Co/SnO_2_, and Co-N/SnO_2_ electrodes, respectively, which means that the phase conversion reaction process of the Co-N/SnO_2_ electrode are significantly prolonged during Li storage. This implies that: 1) the structural stability of Co-N/SnO_2_ electrode is greatly enhanced by the introduction of the 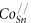 defective states into the SnO_2_ lattice and 2) the particle pulverization process of the Co-N/SnO_2_ sample is successfully delayed during Li insertion, an important indication of the utilization of the plateau capacity above 0.8 V to avoid SEI formation. Furthermore, the overpotential performance of the Co-N/SnO_2_ sample is the smallest one among the three electrodes, which reveals the improvement in electronic/ionic conductivities after Co and N synergistic doping to facilitate Li ion diffusion. On the other hand, the plateau capacity of the Co-N/SnO_2_ electrode is about 540 mAh g^−1^, three times higher with an even lower plateau voltage (0.90 *vs.* 1.55 V) than that of the Li_5_Ti_4_O_12_ spinel anode^34^,^35^ and much higher without SEI formation than that of the commercialized graphite anode (372 mAh g^−1^), making it a promising application for LIBs.

CV measurements were carried out to further clarify the origin of the excellent electrochemical performances of the Co-N/SnO_2_ electrode. Figure 5b,c, and d show the CV curves of the first four cycles of SnO_2_, Co/SnO_2_, and Co-N/SnO_2_ electrodes at a scanning rate of 0.1 mV s^−1^. For all three electrodes, during the first cathodic scanning, the cell exhibits a sharp peak potential at approximately 0.90 V, which corresponds to the structural deterioration of SnO_2_ and the relevant electrolyte decomposition as well^36^. A small cathodic peak at about 0.51 V can be ascribed to the formation of an SEI film, originating from electrolyte decomposition, which is mainly comprised of polymers and insoluble inorganic by-products^37^,^38^. It is clearly shown in Fig. 5b,c, and d that the prominent reduction peaks at about 0.50 V are evidences of the excess formation of SEI film for the Co-N/SnO_2_ electrode, which is probably related to its largest specific surface area (Table S1) and the initial irreversible capacity analysis (Fig. 5a). The structure collapse of SnO_2_ leads to the formation of amorphous Li_2_O matrixes according to the following conversion reaction: SnO_2_ + *x* Li^+^ + *x* e^−^ → Sn^0^ + 2 Li_*x*/2_O. Further, the reversibility of this conversion reaction is still under dispute and needs more precise clarification in the future. The presence of peak potential at about 0.21 V is associated with the alloying reaction in the form of Sn^0^ + *x* Li^+^ + *x* e^-^ ↔ Li_*x*_Sn, 0 ≤ *x* ≤ 4.4, which is attested to be highly reversible during the electrochemical charge-discharge process^36^. During the anodic scan, the peak potential at 0.50 V corresponds to the de-alloying reaction of Li_*x*_Sn products. The formation of large clusters possibly leads to the cracking of electrodes and eventually causes an increase in the contact resistance. In addition, oxidation of metallic Sn^0^ into SnO and further into SnO_2_ could occur when the upper cut-off potential exceeds 800 mV^39^. Nevertheless, the capacities resulting from the oxidations of Sn^0^ to Sn^2+^and Sn^4+^are found to be very small in proportion to the alloying/de-alloying region (in Fig. 5b–d) (Li_*x*_Sn ↔ Sn^0^ + Li_2_O ↔ SnO ↔ SnO_2_). The observed CV profiles are consistent with our previous work^23^ and that of Dahn *et al.*^40^,^41^.

The fitting results give de-alloying peak widths (FWHM) of 0.271, 0.253, and 0.215 V for pure SnO_2_, Co/SnO_2_, and Co-N/SnO_2_ electrodes in the CV cycles, respectively. It is clear that the FWHM value for the Co-N/SnO_2_ electrode is smaller than those of the Co/SnO_2_ and pure SnO_2_ electrodes, which proves that the Co and N co-doping accelerates the electrode reaction process^19^,^20^. As observed in Fig. 5b–d, the other oxidation/reduction peaks also display the same kind of regularity, which helps to explain the superior rate capability of the Co-N/SnO_2_ electrode. On the other hand, it is widely accepted that the difference between redox pairs (D-value) relates to the degree of polarization^42^. Table 2 compares the D-values of pristine, Co/SnO_2_ and Co-N/SnO_2_ electrodes for each alloying/de-alloying redox in the first four cycles. It can be seen that the D-values of the Co-N/SnO_2_ electrode are smaller than those of the pristine and Co/SnO_2_ electrodes, implying that Co and N co-doping effectively decreases the electrode polarization upon cycling.

Figure 5e demonstrates the capacity retentions of pure SnO_2_, Co/SnO_2_, and Co-N/SnO_2_ electrodes at 0.1 C. Undoubtedly the electrode of phase-pure SnO_2_ exhibits fast capacity decay, the discharging capacity of which deteriorates from 1215 to 118 mAh g^−1^ after 50 cycles. In contrast, Co doping and Co/N co-doping can effectively enhance the cycling stability of the SnO_2_ electrode with which the Co-N/SnO_2_ electrode shows obviously optimized electrochemical performance. Specifically speaking, the Co-N/SnO_2_ electrode delivers the highest charging capacity of 1152 mAh g^−1^ in the first cycle, which experiences a gradual capacity decrease within the subsequent 25 cycles. Afterward, it remains almost the same, with a high capacity of about 700 mAh g^−1^ until the 50^th^ cycle (Fig. 5e), the capacity of which is significantly higher than the reported ones^18^,^19^.

After 50 galvanostatic cycles, the rate capabilities of the same electrodes are further evaluated as shown in Fig. 5f. Under the high current densities of 0.2, 1 and 5 C, the Co-N/SnO_2_ electrode could still exhibit desirable capacities of 646, 587, and 557 mAh g^−1^, much higher than the theoretical capacity of a commercialized graphite anode (372 mAh g^−1^). After 90 cycles at various rates ranging from 0.1 to 5 C, a high capacity of 683 mAh g^−1^ could be achieved in the 100^th^ cycle back at 0.1 C, about a 95.4% retention of the capacity at the 50^th^ cycle. This also shows the good cycleability of the Co-N/SnO_2_ electrode. Taking this discussion into account, the remarkably improved electrochemical performances of SnO_2_ after Co/N co-doping, as shown in Fig. 5e and f, can be attributed to a short diffusion path, high crystallization, and good electronic conductivity, as well as the buffering effect of doped Co and N ions in the rutile SnO_2_ lattice.

**Figure 6:**
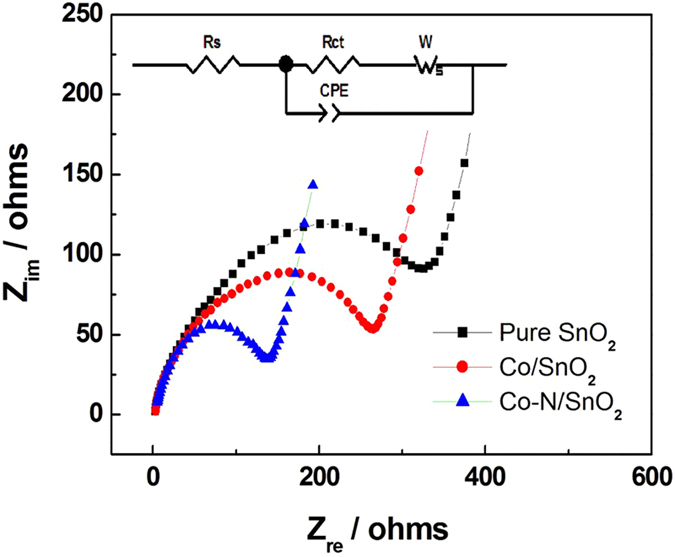
AC impedance spectra of as-assembled SnO_2_, Co/SnO_2_ and Co-N/SnO_2_ cells (inset shows the equivalent circuit).

To elucidate more details to the electrode kinetics process, EIS measurements were carried out using half cells consisting of pure SnO_2_, Co/SnO_2_, and Co-N/SnO_2_ as working electrodes at the state of open circuit voltage (OCV) as demonstrated in Fig. 6. An equivalent circuit (inset of Fig. 6) was used to analyze the obtained impedance spectra. The intercept at the Z_real_ axis at high frequency corresponds to the ohmic resistance (R_s_), which represents the total resistance of the electrolyte, separator, and electrical contacts. The semicircle in the middle frequency range indicates the charge transfer resistance (R_ct_), and the slope region in the low-frequency range represents the Warburg impedance. For more detail, the fitted resistances through the equivalent circuit (Fig. 6) are listed in Table 3. It can be clearly observed that the solution resistances are similar among the three electrodes. Nevertheless, the charge transfer resistance and Warburg impedance are distinctly different among them. The transfer resistance and Warburg impedance are at minimum values of 137.72 and 172.91 Ω for Co-N/SnO_2_ electrode, which are contributed by the co-doping of Co and N into the SnO_2_ nanocrystals.

The conductivity of electrode materials can be calculated with respect to the EIS spectra based on previous reports^43^,^44^, which are derived in the following equation:


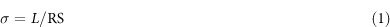


where the parameters σ, L, R, and S represent conductivity, thickness of the electrode materials (45 μm), resistance, and sectional area of the electrode materials (64 mm^2^), respectively. The value of R_ct_ corresponds to the electronic conductivity and that of W_s_ relates to the ionic conductivity, while the total resistance corresponds to the total conductivity. As shown in Table 3, σe is electronic conductivity, σ_i_ is the ionic conductivity, and σ is defined as the total conductivity of the electrode material. The electric conductivity of the pristine material is 4.04 × 10^-5^ Ω^−1^ · cm^−1^, numerically close to that in a previous report^23^, which confirms that our calculation method is reasonable and acceptable. Obviously, the electric conductivity and ionic conductivity of the Co-N/SnO_2_ electrode are 4.07 × 10^−5^ and 2.24 × 10^−5^ Ω^−1^ · cm^−1^, much higher than those of the Co/SnO_2_ and pure SnO_2_ electrode as shown in Table 3. These results are also in turn to support the improved rate capability of the Co-N/SnO_2_ composite since the charge transfer process is the rate-determining step for conversion reactions^45^.

**Figure 7:**
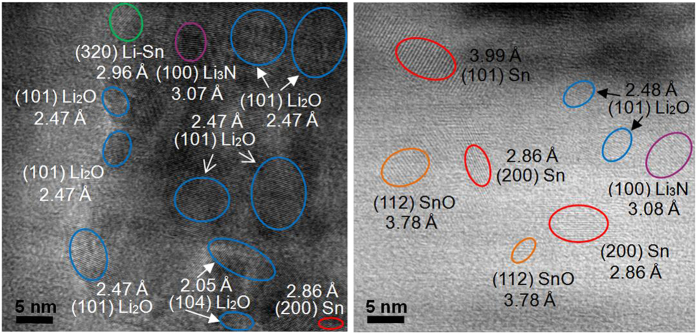
HRTEM images of the Co-N/SnO_2_ electrode. Initially discharged to (**a**) 0.005 V and then charged to (**b**) 3.0 V.

To further elucidate the mechanism of significantly improved electrochemical properties of the Co and N co-doped material, HRTEM is employed to get deep insight into the microstructure evolution at different discharge/charge states. To simplify the observation, the naked Co-N/SnO_2_ powder is used as electrode to assemble cells for cycling. The results by HRTEM observation for the cycled Co-N/SnO_2_ electrodes are shown in Fig. 7. From Fig. 7a, the previous well-crystallized big Co-N/SnO_2_ nanocrystals (~30 nm or larger in Table 1 and Fig. 3) become much smaller after a discharge process to 0.005 V. It can be clearly observed that the pulverization of Co-N/SnO_2_ particles upon lithiation introduces many small ordered domains. The measured *d*-spacings of ordered domains are 3.07, 2.47, 2.05, 2.86 and 2.96 Å, which can be unambiguously assigned to the interplanar distances of (100) for Li_3_N (JCPDS card No. 76-0822), (101) for Li_2_O (JCPDS card No. 77-2144), (104) for Li_2_O (JCPDS card No. 77-2144), (200) for Sn (JCPDS card No. 87-1663) and (320) for Li-Sn (JCPDS card No. 01-0657), respectively. These results supply solid evidences for:1) the Li storage mechanism of first phase conversion (Li_2_O + Sn) and then the alloying reaction (Li_x_Sn) in SnO_2_ electrode; 2) the dispersive distributions of Li-Sn alloys or the intermediate product of Sn nanoparticles into the Li_2_O matrixes, as well as the same conditions for doped Co particles, and 3) the formation of Li_3_N, a good Li ion conductor (approximately 10^−3^ S cm^−1^), which probably comes from the doped N reacting with the inserted Li upon lithiation and obviously have a positive influence on the Li ion transport. Further Fig. 7b shows the HRTEM images of delithiated Co-N/SnO_2_ sample after a reverse charging process to 3.0 V. It can be clearly observed that five kinds of interplanar distances (3.08, 2.48, 3.99, 2.86 and 3.78 Å) are detected in Fig. 7b, which are respectively indexed to the (100) plane of Li_3_N, (101) plane of Li_2_O, (101) plane of Sn, (200) plane of Sn and (112) plane of SnO (JCPDS card No. 88-0287). These results undoubtedly support that the final charging product the SnO_2_ electrode is apt to SnO nanoparticles and the further oxidation of Sn^2+^ to Sn^4+^ probably becomes much more difficult without any energy gain. Based on the widely accepted reaction mechanism of SnO_2_ electrode, it is not surprising to observe the existence of Li_2_O, Sn, and Li-Sn in the discharged state and Li_2_O, Sn and SnO in the charged state, which are in accordance with the CV analysis of Fig. 5d. Due to its poor decomposing kinetics in LIBs, the *d*-spacing of the new product of Li_3_N, still can be found even at 3.0 V during the charging of SnO_2_ electrode at the rate of C/10, which is in consistence with the previous reports^46^,^47^,^48^,^49^. Thus in turn, the dispersed Li_3_N herein contributes a lot to the Li ion diffusion kinetic of the whole Co-N/SnO_2_ electrode of in electrochemical cycling. This should play an important role in promoting the ionic conductivity and rate performance of Co-N/SnO_2_ electrode as discussed previous in Fig. 5f and Table 3.

## Conclusions

In summary, the Co-N doped SnO_2_ nanocrystals have been synthesized and further employed as anode to investigate the Li storage properties for LIBs. The as-prepared Co and N co-doped sample exhibits better capacity retention and rate capability compared with pure and Co doped materials. The excellent electrochemical performances can be attributed to the synergistic effects of 1) reduced particle size and buffering effect of the doping elements Co and N to afford the volume change during cycling and 2) increases in the crystallinity, BET surface area, pore diameter and conductivity to facilitate the diffusion of lithium ions. As a result, this Co-N/SnO_2_ nanocrystal electrode demonstrates significantly improved cycleability and rate performance. These findings provide significant guidance for designing anode materials with higher reversible capacities for LIBs. Moreover, this approach could be widely applied to large-scale production of nanostructured powders with various compositions and a wide variety of applications.

## Experimental

### Material Synthesis

The synthesis of the Co/SnO_2_ samples was performed using water as a solvent, starting from SnCl_4_ · 5H_2_O, Co(NO_3_)_2_ · 6H_2_O and NaOH. Typically, 20 ml of SnCl_4_ (4 mmol) and Co(NO_3_)_2_ (0.24 mmol) solution was added to 20 ml of NaOH (28 mmol) with vigorous stirring (Co/SnO_2_ = 6/100 *wt.*%). The obtained solution was then transferred into a 50 ml Teflon-lined autoclave and maintained at 180 °C for about 12 h. Afterward, the flaxen precipitation was collected, washed with deionized water and ethanol to remove impurities, and subsequently dried in a vacuum oven at 70 °C. The phase pure SnO_2_ nanocrystal was obtained through the same route. To prepare Co and N co-doped SnO_2_, the obtained Co/SnO_2_ was further sintered at 500 °C for 12 h under nitrogen atmosphere resulting in residual nitrate decomposition and N elements doping into Co/SnO_2_ material.

### Physical Characterizations

X-ray diffraction (XRD) patterns were recorded on a DX-2500 diffractometer (Fangyuan, Dandong) with Cu Kα radiation of λ = 0.154145 nm. The Raman spectra were collected on a laser Raman spectrometer (RM-1000, Renishaw) with a 633 nm He-Ne laser. Morphological characterization was carried out by field-emission scanning electron microscopy (FESEM) (JEOL JSM 7001F). Energy dispersive X-ray analysis (EDAX) was applied to determine the element composition and distribution, together with FESEM. The images of high-resolution transmission electron microscopy (HRTEM) were collected on Philips CM200 equipment to investigate the pristine material and electrode microstructures. Nitrogen adsorption measurements were performed on a Micromeritics ASAP 2020 adsorption analyzer. Specific surface areas were calculated by the Brunaure-Emmert-Teller (BET) method. Pore volumes and sizes were estimated from pore size distribution curves from the adsorption isotherms using the Barrett-Joyner-Halenda (BJH) method. X-ray photoelectron spectroscopy (XPS) was performed on a Thermo Electron Corporation spectrometer with an Al Kα (1486.6 eV) radiation.

### Electrochemical Characterizations

The working electrodes were fabricated by spreading the slurry of 80 *wt.*% active material (SnO_2_, Co/SnO_2_, Co-N/SnO_2_), 10 *wt.*% acetylene black (Super-P, MMM Carbon), and 10 *wt.*% binder (polyvinylidene fluoride, PVDF) dissolved in N-methyl-pyrolline (NMP) onto the copper foil. After being thoroughly dried, the films were cut into squares with 8 mm in length as working electrodes. The corresponding loading weight was about 1.64 mg cm^−2^. The electrolyte was 1 **M** LiPF_6_ dissolved in a mixture of ethylene carbonate (EC) and dimethyl carbonate (DMC) with a volume ratio of 1:1. The assembly process was conducted in an argon-filled glove-box with the content of H_2_O and O_2_ less than 1 ppm.

Gavalnostatic cycling was measured on a battery charger (LAND BT1-10, China) between 0.005 and 3.0 V (*vs.* Li/Li^+^) at 0.1 C. Cyclic voltammetry (CV) profiles were performed on an electrochemical workstation with a three-electrode system (CHI660D, Shanghai Chenhua). The CV curves were recorded between 0.005 and 3.0 V at a scanning rate of 0.10 mV s^−1^. Electrochemical impedance spectroscopy (EIS) measurements were performed over a frequency range from 100 kHz to 10 mHz.

## Additional Information

**How to cite this article**: Wan, N. *et al.* Improved Li storage performance in SnO_2_ nanocrystals by a synergetic doping. *Sci. Rep.* **6**, 18978; doi: 10.1038/srep18978 (2016).

## Supplementary Material

Supplementary Information

## Figures and Tables

**Table 1:** Lattice parameters and grain sizes of pure SnO_2_, Co/SnO_2_ and Co-N/SnO_2_ samples.

Samples	*a*/Å	*c*/Å	grain size/nm
SnO_2_	4.7655	3.1843	34.0
Co/SnO_2_	4.7751	3.1854	35.6
Co-N/SnO_2_	4.7973	3.1993	39.2

**Table 2:** Redox potentials and D-values of pristine, Co/SnO_2_ and Co-N/SnO_2_.

Cycle number	Redox potential/V			D-Value/V^a^		
	Pristine	Co/SnO_2_	Co-N/SnO_2_	Pristine	Co/SnO_2_	Co-N/SnO_2_
1^st^	0.52/0.19	0.53/0.18	0.51/0.21	0.33	0.35	0.30
2^nd^	0.52/0.18	0.52/0.18	0.48/0.22	0.34	0.34	0.26
4^th^	0.50/0.17	0.51/0.19	0.47/0.23	0.33	0.32	0.24

^a^D-value: difference between redox pairs.

**Table 3:** Fitting results of EIS data for pure SnO_2_, Co/SnO_2_, and Co-N/SnO_2_.

Sample	R_s_ (Ω)	R_ct_ (Ω)	W_s_ (Ω)	σ_e_ (10^-5^Ω^−1^·cm^−1^)	σ_i_ (10^−5^Ω^−1^·cm^−1^)	R_tol_ (Ω)	σ (10^−5^Ω^−1^·cm^−1^)
Pure SnO_2_	2.90	327.68	325.12	2.15	2.16	655.70	1.07
Co/SnO_2_	2.84	264.27	235.24	2.67	2.99	502.35	1.40
Co-N/SnO_2_	3.08	137.72	172.91	5.11	4.07	313.71	2.24
