# Supplementary material for: Improved Li storage performance in SnO2 nanocrystals by a synergetic doping
Source: Sci Rep. 2016 Jan 6;6:18978. doi: 10.1038/srep18978 (PMC4702176; doi:10.1038/srep18978)
Supplement: Supplementary Information [file srep18978-s1.doc]

**Electronic Supplementary Data**

**Improved Li storage performance in SnO_2_ nanocrystals by a synergetic doping**

Ning Wan^1^, Xia Lu^2^, Yuesheng Wang^3^, Weifeng Zhang^1^, Ying Bai^1,4,*^, Yong-Sheng Hu^3^, Sheng Dai^4^

^1^ Key Laboratory of Photovoltaic Materials of Henan Province and School of Physics & Electronics, Henan University, Kaifeng 475004, PR China.

^2^ Materials Engineering, McGill University, Montréal (Québec) H3A 0C5, Canada.

^3^ Institute of Physics, Chinese Academy of Sciences, Beijing 100190, PR China.

^4^ Chemical Sciences Division, Oak Ridge National Laboratory, Oak Ridge, TN 37831, USA.

^*^Corresponding author. Tel.: +86-0371-23881602；E-mail address: ybai@henu.edu.cn


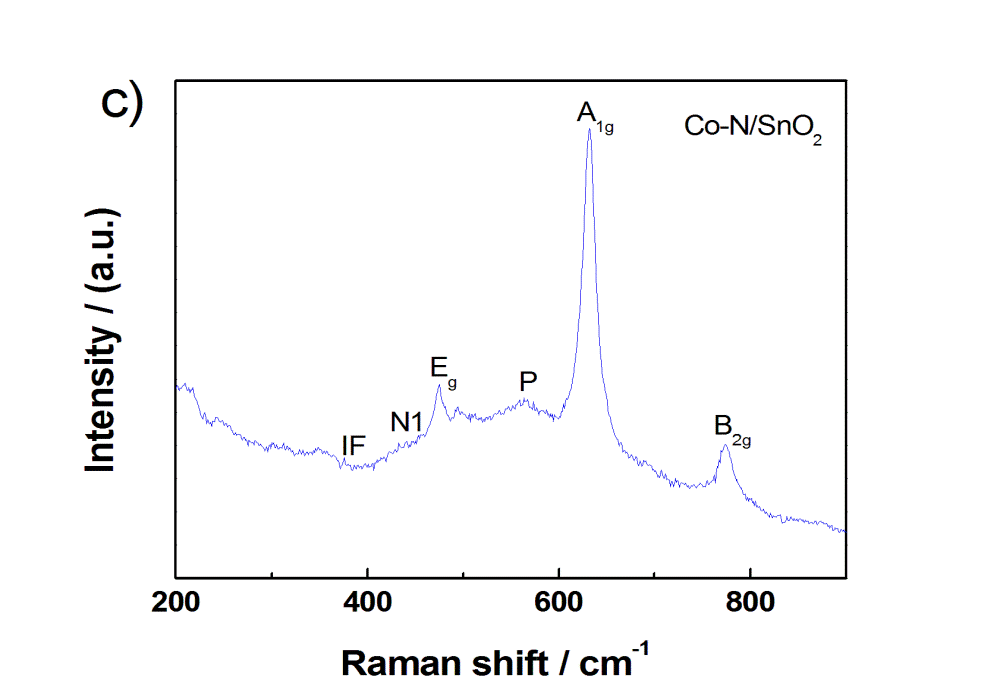

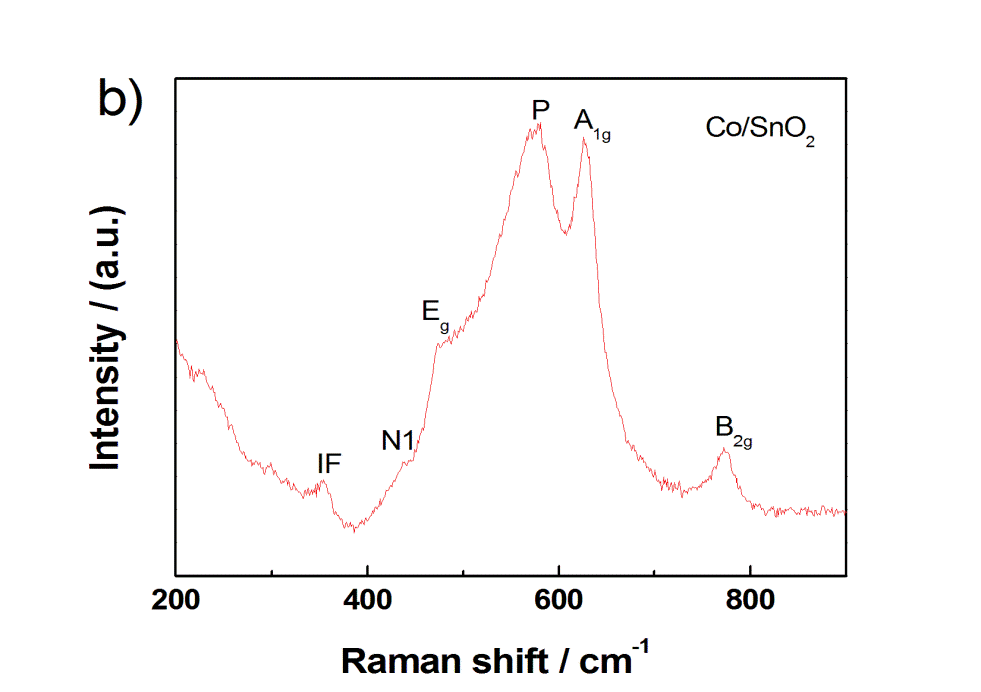

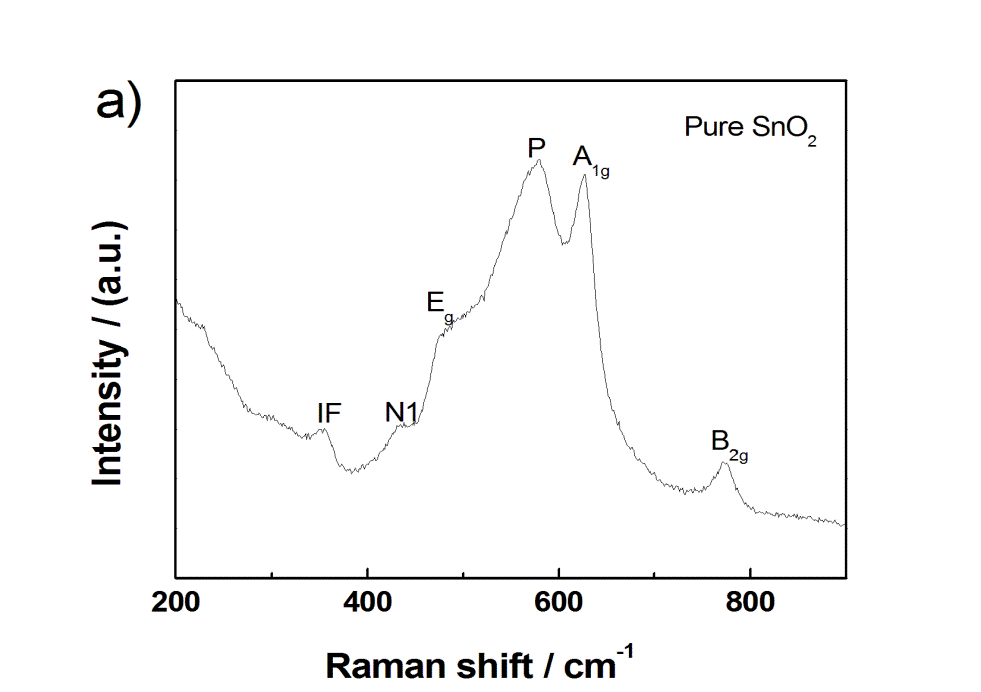


**Figure S1** Raman spectra of pure SnO_2_, Co/SnO_2_ and Co-N/SnO_2_ samples.


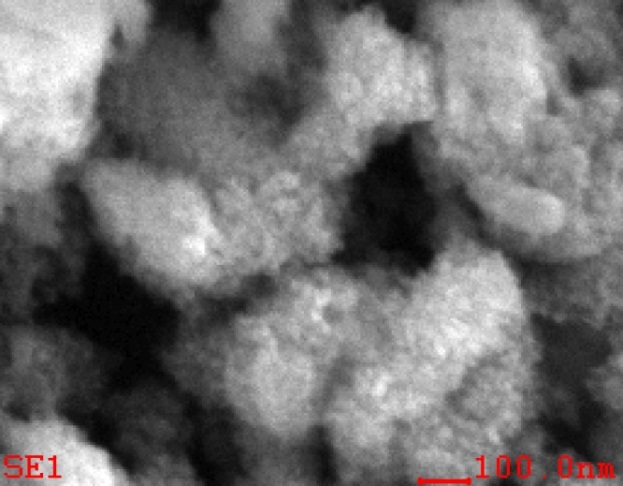


**200nm**

a)


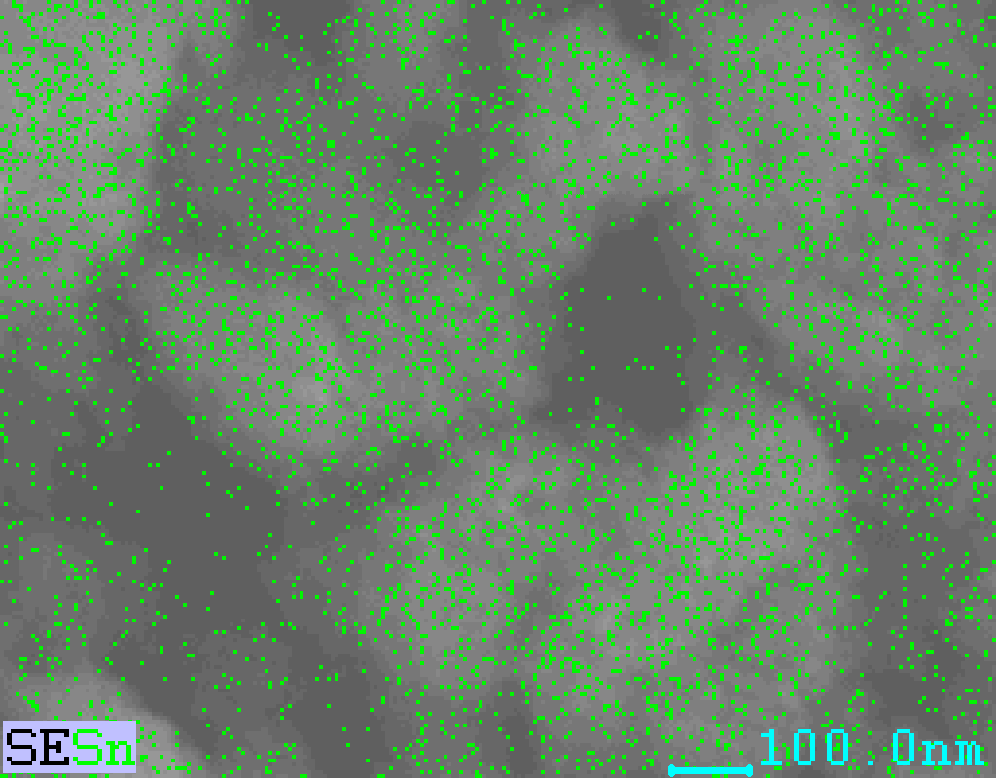


Sn-L

d)


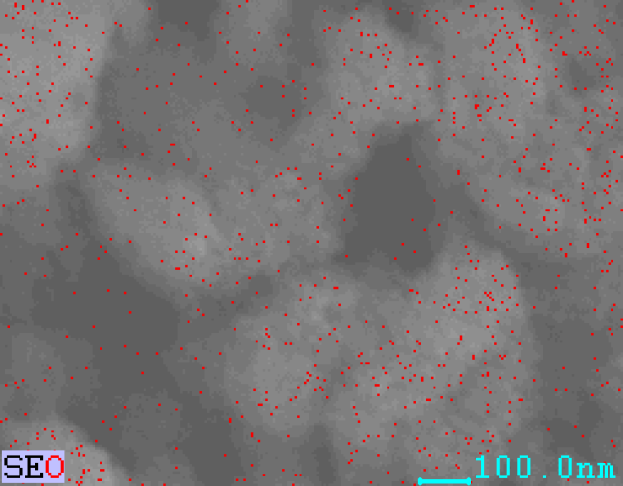


O-K

c)


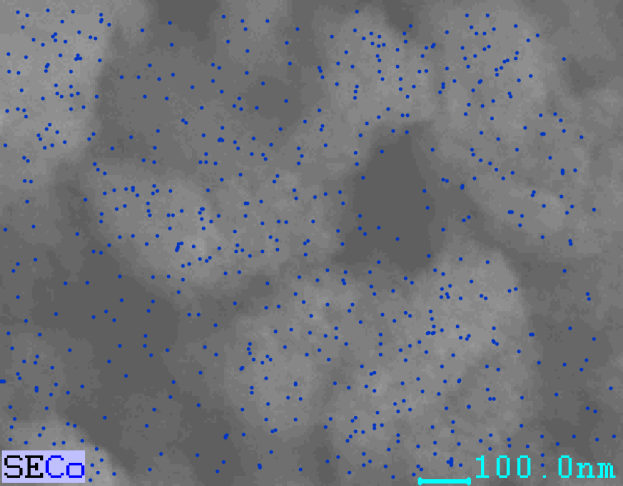


Co-K

e)


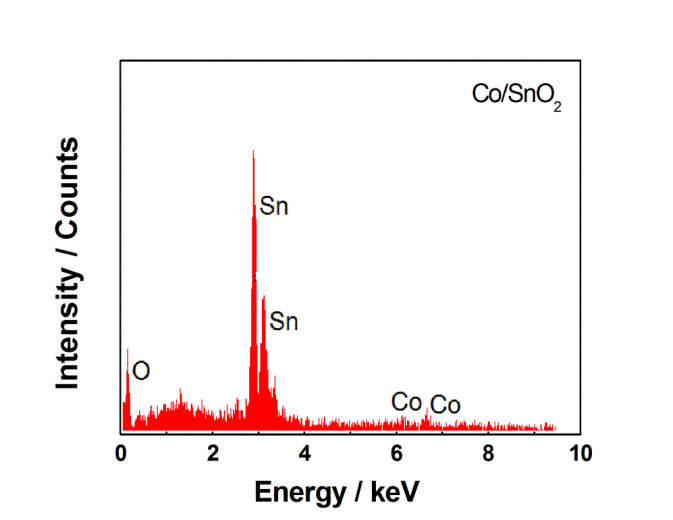


b)


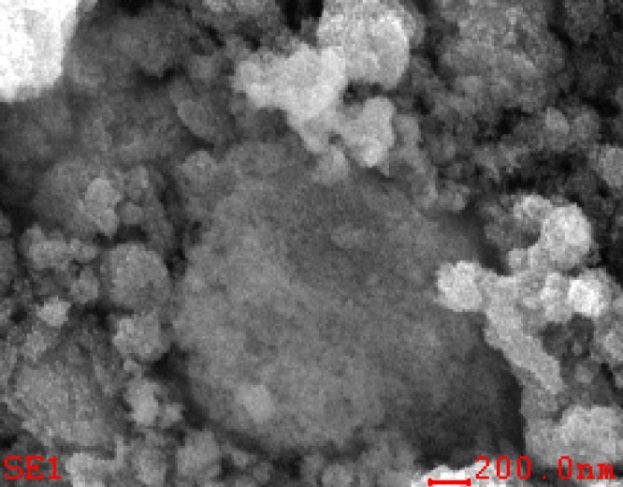


**200nm**

f)


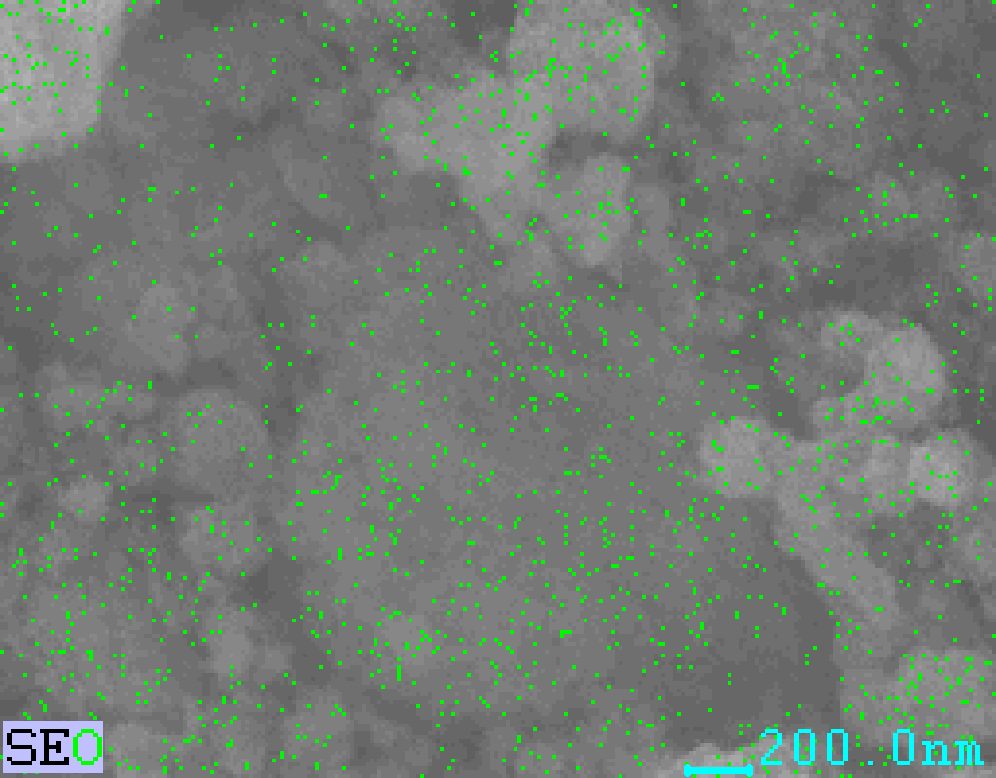


O-K

h)


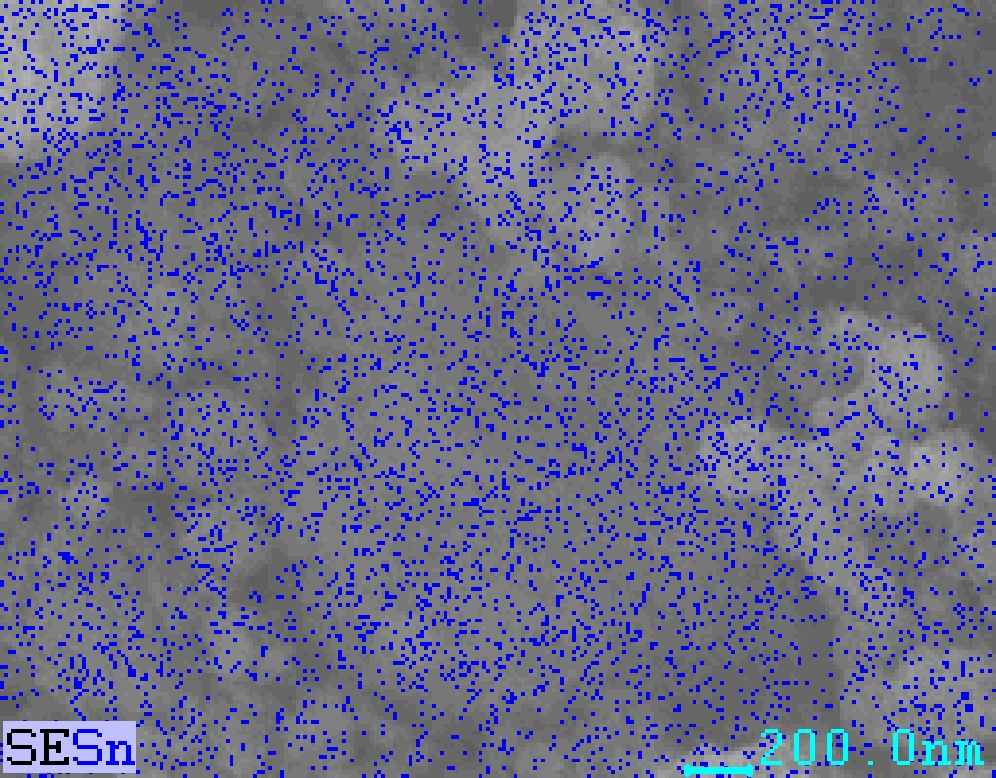


Sn-L

i)


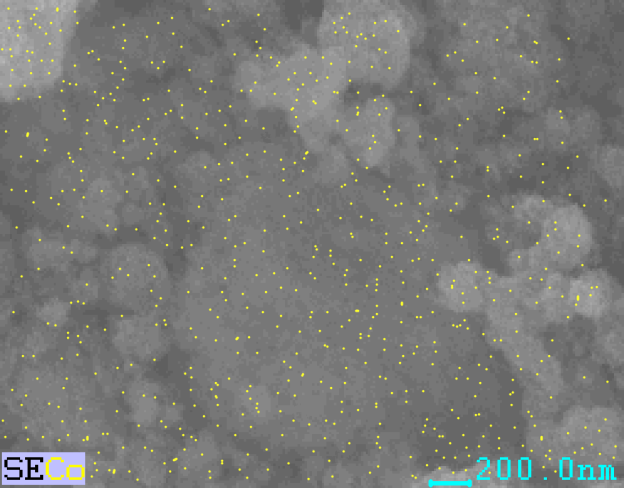


Co-K

j)


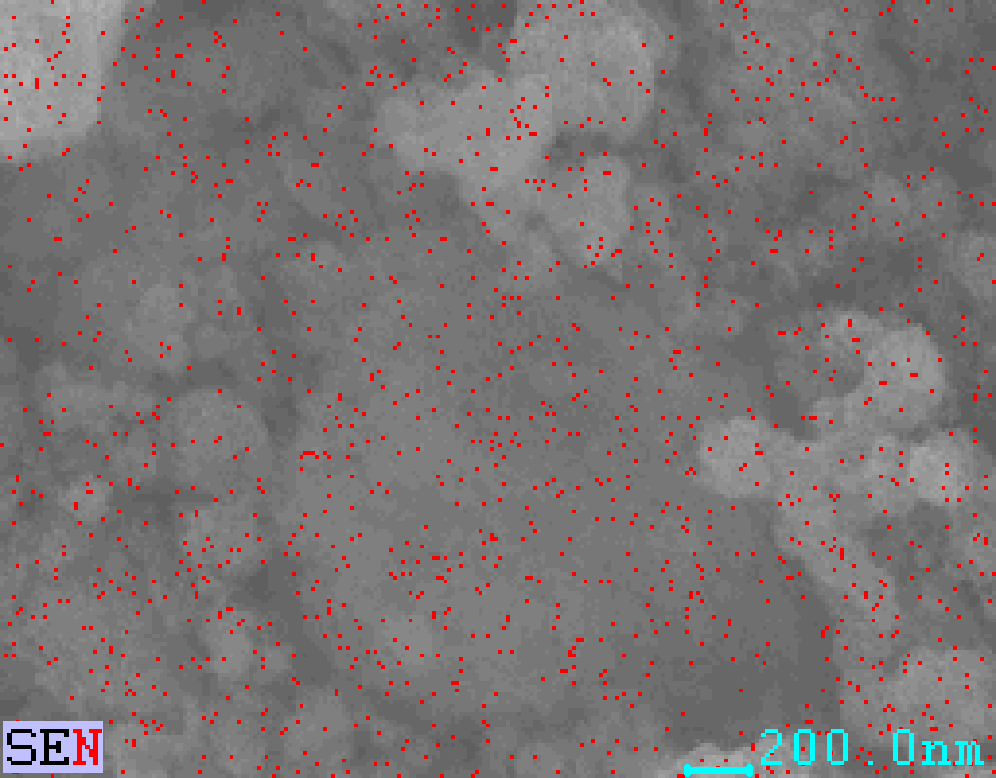


N-K

k)


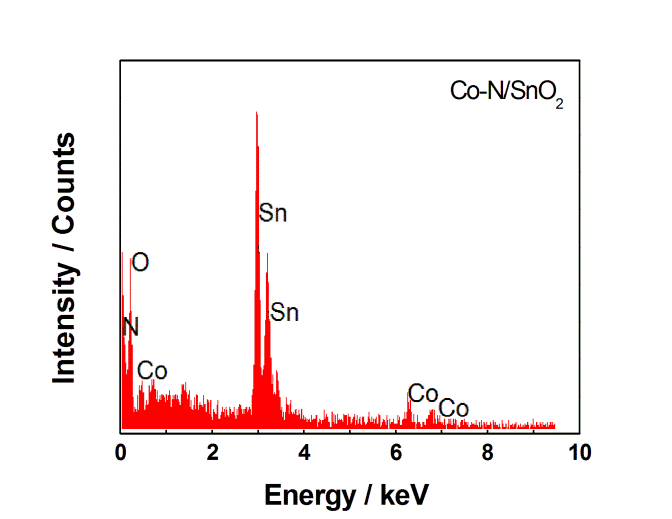


g)

**Figure S2** SEM image (a), EDAX spectrum (b), and oxygen, tin and cobalt (c-e) element mapping images of Co/SnO_2_. SEM image (f), EDAX spectrum (g), and oxygen, tin, cobalt and nitrogen (h-k) element mapping images of the Co-N/SnO_2_.

**Table S1** Specific surface area, pore volume and average pore size of the different samples.

| **Samples** |  | **SBET (m^2^ g^-1^)** |  | **Pore volume (cm^3^)** |  | **Average pore size (nm)** |
| --- | --- | --- | --- | --- | --- | --- |
| SnO_2_ |  | 123 |  | 0.030 |  | 8.7 |
| Co/SnO_2_ |  | 130 |  | 0.037 |  | 8.9 |
| Co-N/SnO_2_ |  | 139 |  | 0.042 |  | 9.1 |
